# RETRACTION: Nephrolithiasis: Molecular Mechanism of Renal Stone Formation and the Critical Role Played by Modulators

**DOI:** 10.1155/bmri/9842796

**Published:** 2025-09-15

**Authors:** BioMed Research International

RETRACTION: K. P. Aggarwal, S. Narula, M. Kakkar, and C. Tandon, “Nephrolithiasis: Molecular Mechanism of Renal Stone Formation and the Critical Role Played by Modulators,” *BioMed Research International* 2013, (2013): 292953, https://doi.org/10.1155/2013/292953.

The above article, published online on 14 September 2013 in Wiley Online Library (http://wileyonlinelibrary.com), has been retracted by agreement between the Editorial Board and John Wiley & Sons Ltd. The retraction has been agreed due to substantial textual overlap with multiple sources [1–4].

The authors did not respond to the retraction.

## References

[1] Khan S. R. and Kok D. J. , Modulators of Urinary Stone Formation, Frontiers in Bioscience. (2004) 9, no. 1-3, 1450–1482, 10.2741/1347, 2-s2.0-2442698061. 14977559

[2] Khan S. R. and Kok D. J. , Stoller M. and Meng M. , Modulators of Crystallization of Stone Salts, Urinary Stone Disease, 2007, Current Clinical Urology, Humana Press, Springer Nature, 175–219, 10.1007/978-1-59259-972-1_10.

[3] Tsujihata M. , Mechanism of Calcium Oxalate Renal Stone Formation and Renal Tubular Cell Injury, International Journal of Urology. (2008) 15, no. 2, 115–120, 10.1111/j.1442-2042.2007.01953.x, 2-s2.0-38849085850. 18269444

[4] Verkoelen C. F. , Crystal Retention in Renal Stone Disease: A Crucial Role for the Glycosaminoglycan Hyaluronan?, Journal of the American Society of Nephrology. (2006) 17, no. 6, 1673–1687, 10.1681/ASN.2006010088, 2-s2.0-33646906175. 16707562

